# Effect of plant and microalgae on immune-related genes and resistance of nile tilapia (*Oreochromis niloticus*) against *Aeromonas hydrophila*

**DOI:** 10.1038/s41598-025-09715-3

**Published:** 2025-07-29

**Authors:** Tasneem Ahmed Abdelmohsen, Adel Hassan Saad, Rasha A. Al wakeel, Sabreen E. Fadl, Awatef Hamed Hamouda

**Affiliations:** 1 Department of Fish Health and Diseases, Faculty of Fish and Fisheries Technology, Aswan University, Aswan, 81528 Egypt; 2 Nutrition and Clinical Nutrition Department, Faculty of Veterinary Medicine, Matrouh University, Matrouh, 51744 Egypt; 3 Department of Physiology, Faculty of Veterinary Medicine, Kafrelsheikh University, Kafrelsheikh, Egypt; 4 Biochemistry Department, Faculty of Veterinary Medicine, Matrouh University, Matrouh, Egypt

**Keywords:** Nile tilapia, Lemongrass, *Arthrospira platensis*, Growth performance, Gene expression, Biochemistry, Physiology

## Abstract

Nile tilapia, is a crucial fish for warmwater aquaculture globally and plays a key role in food security. The experiment’s goal was to ascertain the actual effects of lemongrass and/or *Arthrospira platensis (A. platensis)* on *Oreochromis niloticus (O. niloticus)* growth. Along with liver and kidney functioning, some organ histopathology, and the expression of genes linked to immunity in liver tissue both before and after an infection with *Aeromonas hydrophila (A. hydrophila)*. There were four diets created: with the first serving as a control group and being additive-free, while the second included 7.5 g kg^− 1^ of diet of *A. platensis*, the third included 200 mg kg^− 1^ of diet of lemongrass (*Cymbopogon citratus*), and the fourth group included lemongrass and *A. platensis*. The results of the growth trail were significantly higher in the treatment groups, especially with *A. platensis* treatment. However, the results of the biochemistry and immunological assay were pronounced with improved intestinal morphometry in the combination groups without pathological lesions. Moreover, the antioxidant and proinflammatory cytokine gene expressions improved with the treatments used. After experimental challenges, the results of the treatment groups showed the immunomodulatory effects of *A. platensis* and lemongrass oil against *A. hydrophila* infection. Thus, *A. platensis* alone or with lemongrass improved growth performance before experimental infection. After experimental infection, they decreased mortality rate and pathological lesions in liver and spleen and improved biochemical parameters and antioxidant and proinflammatory cytokine gene expression.

## Introduction

In addition to providing jobs and revenue, aquaculture is a significant economic activity on a global scale, currently accounting for 50% of fish consumption^1^. However, disease-related issues have caused economic losses to the world’s aquaculture output that are estimated to be between 1.05 and 9.58 US billion dollars per year^2^. Nowadays, the plants and algae are used to improve aquaculture through increasing growth and immunity^3^.

lemongrass essential oil (LEO) is a volatile oil that can be extracted straight from fresh lemongrass (*Cymbopogon citratus*). The grass has an essential oil content of 0.035%^4^. The primary ingredient in LEO, citral, is well-known for its fungistatic, anti-inflammatory, antiseptic, immunomodulatory, and antibacterial qualities^5^. Moreover, other phytoconstituents such as menthone, geranial, trans-caryophyllene, and delta-3-carene are present in LEO^4^. Because of its antibacterial properties, LEO can be used in fish aquaculture in place of antibiotics^6,7^. Lemongrass oil has antioxidant activity and a high vitamin C content^8^. Although LEO had no effect on fish survival following an infection with *A. hydrophila*, 0.50 ml/kg diet might enhance growth and haematological parameters in tambaqui^9^. Moreover, lemongrass is widely utilized in the cosmetics, perfume, and food processing industries as a food flavoring^10^. Alasgah et al.^11^ found that LEO might prevent the formation of V. parahaemolyticus biofilms and improve the safety of fish that are eaten.

*Arthrospira platensis (A. platensis)* is the most popular cultivated microalga being produced commercially^12^. *Arthrospira platensis* is a type of algae that is blue-green in color and grows in oceans and salty lakes in subtropical climates. It is a type of *Cyanobacteria* that has an unusually high protein content (55–70% by dry weight); it contains all of the essential amino acids and matches the profile of a well-balanced reference protein provided by the Food and Agriculture Organization/World Health Organization^13^. With its many uses in aquatic systems and ecosystem management, the adaptable microalga *A. platensis* has become a potent instrument for advancing environmental sustainability^14^. *Arthrospira platensis* is a superfood due to its richness in plant pigments and its ability to regulate photosynthesis, which has made it a popular ingredient in nutritional supplements. *Arthrospira platensis* has been used as a partial or complete replacement for protein in fish feed for a variety of fish species, including Nile tilapia^15^, great sturgeon^16^, rainbow trout^17^, olive flounder^18^, parrot fish^19^, catfish^20^, goldfish^21^, mrigal carp^22^, and guppy^23^. However, *A. platensis* is a major contributor to sustainability in the food and health industries due to its dual nature as an economical and environmentally benign solution for carbon neutrality and bioproduct creation^24^.

Despite all this research, it still lacks focus on the effect of *A. platensis* on the most important and economically important species of fish. *Arthrospira platensis* has drawn more and more attention lately as a possible option for treating wastewater, especially fish culture effluent^25^. *Arthrospira platensis* appears to be a very good integrated strategy for fish farming when used as a nutritional supplement and to treat effluent for water quality control.

While reducing the use of traditional synthetic chemotherapeutics—many of which are prohibited in various countries owing to the dangers of toxicity to fish and handlers as well as environmental contamination—it is imperative to pursue technical advancements in order to produce fish products of higher quality^1^. Thus, in order to improve the activity’s overall sustainability, contemporary research has concentrated on natural, non-toxic, and eco-friendly therapies. So, the *A. platensis* and lemongrass alone or in combination were used to improve performance and immunity against experimental infection.

## Materials and methods

### Feed additives

Lemongrass was used as oil, contributed by the Faculty of Agriculture, Aswan University, Aswan, Egypt. Meanwhile, dried *Arthrospira platensis* (*A. platensis*) granules from the Cyanobacteria Research Lab at the Sakha Agriculture Research Station in Kafrelsheikh, Egypt, were utilized.

### Utilizing fish and designing an experiment

Mono sex *O. niloticus* (49.25 ± 0.5 g) fish (45 fish/group, each in three glass aquaria (80 х 30 х 45 cm)) were randomly assigned to perform this study (*n* = 180). Fish were introduced from a local farm owned by High Dam Lake Development Authority in Aswan Governorate to the Faculty of Fish and Fisheries Technology, Aswan University, Aswan, Egypt, which approved (2/2023) this study and acclimatized for 14 days. During acclimatization, fish fed on control experimental diet 30% protein. Four diets with similar quantities of calories and 30% protein each were created (Table 1). The first serving as a control group and being additive-free, while the second (SP) included 7.5 g kg^− 1^ of diet of *A. platensis*^26^, the third (LG) included 200 mg kg^− 1^ of diet of lemongrass *(Cymbopogon citratus)*^27^, and the fourth (LG + SP) group included lemongrass at 200 mg kg^− 1^ pulse 7.5 g kg^− 1^ of *A. platensis*. For two months, fish feed twice a day at a rate of 3%. The dechlorinated water was used to replace 30% of the aquarium water each day. The water oxygen, salinity, and temperature were adjusted at 5.8–6.1 ppm, 1.1-2‰, pH 7.4–8.1, and 24 °C, respectively. All methods were performed in accordance with the relevant guidelines and regulations.

**Table 1:** Physical and chemical composition of basal diet.

Formulation	Basal diet
	g
Fish meal (60% cp.)	178.5
Soybean meal (44% cp.)	378.9
Meat meal (55%)	13.1
Wheat middling	195
Rice polishing	177.3
Fat Soyabean oil	25.7
Lime stone	8.7
Vitamin and mineral mix	9.3
Di-calcium phosphate	2.2
NaCl	4.3
Antimycotoxin	5.9
Vitamin premix E	1.1
Crude protein	33.67
Lipid	8.4
Fibers	6.3
Ash	11.8
Gross energy (kJ/g)	19.06

*CP* crude protein.

The growth performance of the experimented fish was calculated every 2 weeks. A full day prior to the sampling, fish was fasted. Fish was anaesthetized with tricaine methane sulphonate (MS-222) at a dose of 25 mg/L prior to sampling. On days 60 (end of the growth trial) and 78 (7 days after *A. hydrophila* infection) of the experiment, blood was collected from the caudal vein with anticoagulant (for hemogram and leukogram) and without anticoagulant (for liver and kidney functions) to separate serum (9 fish/group, three/replicate). The phagocytic activity and index were determined according to the following equations:

$${\text{Phagocytic activity}}\,=\,{\text{macrophages containing yeast}}/{\text{total number of macrophages }} \times {\text{1}}00,$$

$${\text{phagocytic index}}\,=\,{\text{number of cells phagocytized}}/{\text{ number of phagocytic cells}}.$$

It was there that the number of phagocytic cells was counted.

When gathering data, the researchers were not blinded. Analysis was done using blinding.

### An analysis of intestinal morphometry and histopathology

Liver, intestine, spleen, and gills sections were prepared for pathological assessment using the standard paraffin embedding method after being promptly fixed in 10% formalin. For light microscopic analysis, sections with a thickness of 5 μm were cut and stained with hematoxylin and eosin (HE)^28^. Villi length and width and intravilli space were measured as part of the intestinal morphometry.

### Gene expression

Tissue samples (liver) were taken from five fish per replicate (15 fish/group) after euthanizing in a bath of clove oil. The samples were gathered in liquid nitrogen and stored at − 80 °C until used. The total *RNA* was extracted from each sample using Trizol (Applied Biotechnology, Egypt) according to the accompanied Kit instructions. The integrity of the extracted *RNA* was inspected using ethidium bromide-stained agarose gel electrophoresis. The *mRNA* was reverse transcribed backward to the *cDNA* using a specific kit (Applied Biotechnology, Egypt).

### Real-time PCR

The gene thermal amplification was done by means of the Stratagene MX3000P real-time PCR system using the Low-Rox-SYBR green kits (Bioline, UK). The amplification conditions were done according to Esam et al.^29^, and the specific annealing temperatures for each gene are listed in Table 2.

**Table 2:** The primers used in this study.

Gene	Primer	Accession no	References
B actin	F: CAGCAAGCAGGAGTACGATGAG R: TGTGTGGTGTGTGGTTGTTTTG	XM_003455949.2	El-Kassas et al. (2020)^98^
*il-8*	F: CTGTGAAGGCATGGGTGTGGAG R: TCGCAGTGGGAGTTGGGAAGAA	NM_001279704.1	Abdo et al. (2022)^99^
*il-1β*	F: TCAGTTCACCAGCAGGGATG R: GACAGATAGAGGTTTGTGCC	XM_019365842.1	
*nfkb2*	F: GAACATCAGACCGACGACCA R: TCTCCGCCAGTTTCTTCCA	XM_003457469.4	
*tnf-α*	F: AAGCCAAGGCAGCCATCCAT R: TTGACCATTCCTCCACTCCAGA	NM_001279533.1	Esam et al. (2022)^29^
*Cat*	F: CCCAGCTCTTCATCCAGAAAC R: GCCTCCGCATTGTACTTCTT	JF801726.1	Abdo et al. (2021)^100^
*Gpx*	F: CCAAGAGAACTGCAAGAACGA R: CAGGACACGTCATTCCTACAC	DQ355022	El-Kassas et al. (2022)^101^

*β-actin* housekeeping gene, *nf-κb2* Nuclear factor kappa B, *il-1β* Interleukin 1β, *il-8* Interleukin 8, *cat* catalase, *gpx* glutathione peroxidase.

The gene expression levels in pre challenged and post challenged groups were calculated as a fold change (2^−ΔΔCT^) according to Livak and Schmittgen^30^. Where the data were normalized against the cycle threshold of the control group (fed a basal diet and not infected). The β-actin was chosen as a control to normalize the expression levels of the genes.

### *Aeromonas hydrophila* infection

After the end of the growth trial, 20, 15, 15, and 15 fish from the control, *A. platensis*, lemongrass, and mixed groups were challenged (0.2 ml dose of 24 h.) intraperitoneally (IP) with the pathogenic strain of *Aeromonas hydrophila*. The pathogenic strain was acquired from Kafrelsheikh University in Egypt, Department of Fish Diseases, Faculty of Veterinary Medicine. *A. hydrophila* concentration (1 × 10^8^ cells/ml) was adjusted to the density of McFarland Standard 1^31^. According to Amos^32^, fish were observed for 7 days to record the clinical signs and mortality rate.

The blood and tissue samples were collected after 60 days to ensure that the treatment affects growth performance, where the minimum period for a growth trial is 60 days. Moreover, the samples were collected after 7 days from experimental infection to avoid the effect of stress as a result of the handling of fish during experimental infection and to ensure the results were produced due to experimental infection and didn’t increase the period to avoid the death of fish as a result of infection.

### Statistical analysis

The minimum sample size required for a research study was ascertained by power analysis prior to the experiment’s commencement. Additionally, in order to demonstrate homoscedasticity and normality, the data were subjected to the Shapiro-Wilk and Levene tests for normal distribution. Next, using SPSS version 20, a one-way analysis of variance was used to statistically analyze the data.

## Results

### Clinical signs

**Fig. 1:**
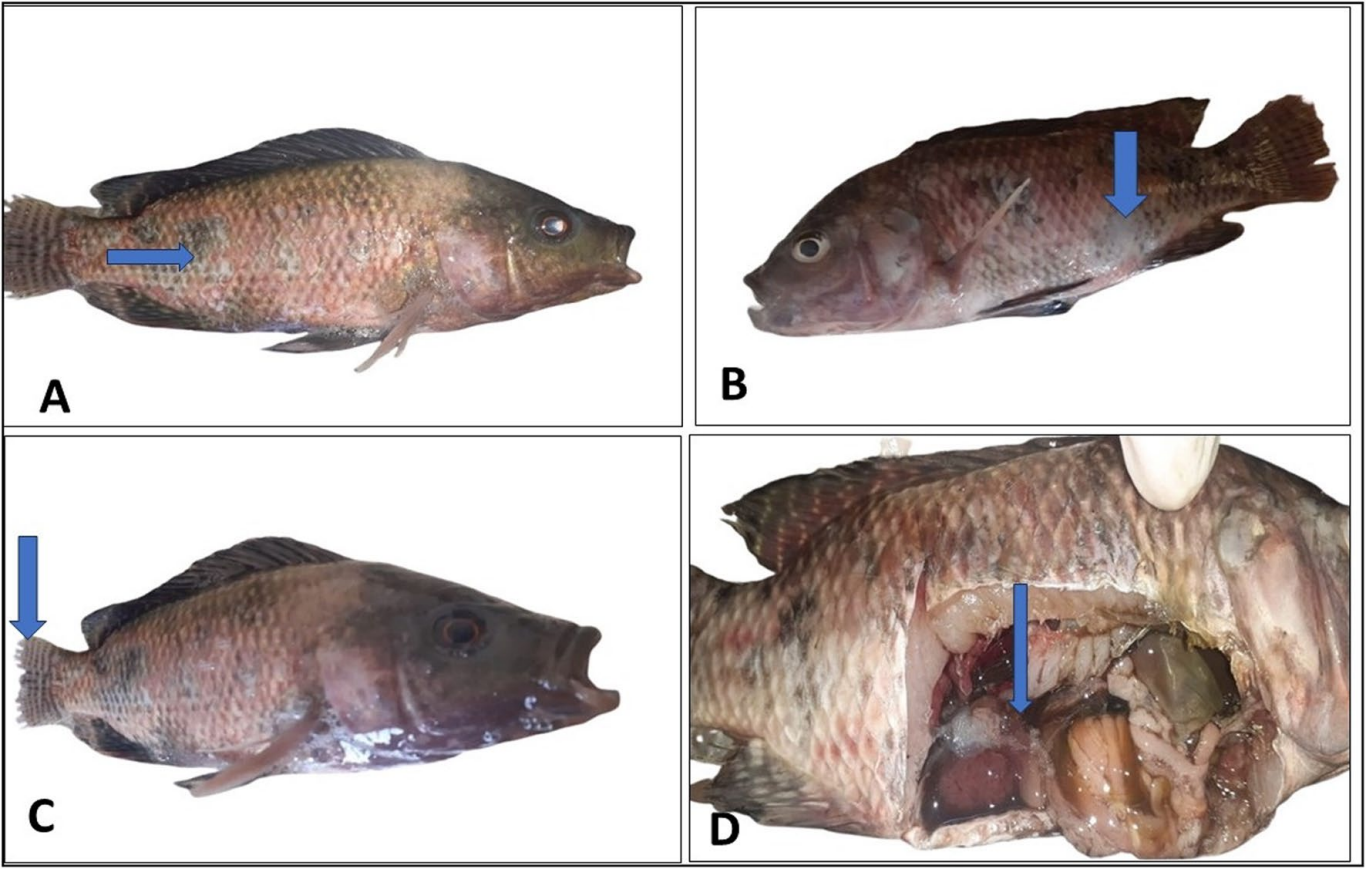
Displayed the postmortem lesion and clinical symptoms of the artificially infected fish.

The mortality of fish following an experimental *A. hydrophila* infection continued for five days, then stopped (Table 3). Compared to the SP *platensis*, LG, and LG + SP groups, the control group’s mortality rate increased significantly (*P* ≤ 0.05). Furthermore, the SP, LG, and LG + SP groups’ survival rates were considerably (*P* ≤ 0.05) higher than those of the control group. The fish started dying after days of infection. The clinical symptoms appeared in skin ulcers (Fig. 1A, B), tail erosions (Fig. 1c). Postmortem, there were enlarged liver, spleen, and gall bladder with abdominal edema (Fig. 1D).

**Table 3:** Experimental infection of nile tilapia with *Aeromonas hydrophila* after growth trial.

Parameters	Groups			
	Control	SP	LG	LG + SP
Total number	15	10	10	15
Dead number	10	2	2	2
Survival rate	1.67 ± 0.33^b^	4 ± 0.58^a^	4 ± 0.0^a^	4.33 ± 0.33^a^
Mortality rate	3.33 ± 0.33^a^	1 ± 0.57^b^	1 ± 0.0^b^	0.67 ± 0.33^b^

Values are expressed as mean ± standard errors. Means in the same row (a-c) with different letters significantly differ at (*P* ≤ 0.05).

### Growth

The leverage of lemongrass and/or *A. platensis* on the performance of Nile tilapia is shown in Table 4. The statistical analysis of the collected data on the final body weight, weight gain, and FCR of the SP group significantly (*P* < 0.05) improved in contrast to the other groupings. However, as compared to the control group, the LG and LG + SP groups showed improvements in the same parameters.

**Table 4:** Impact of *A. platensis* and Lemongrass on nile tilapia growth performance after two months (*n* = 45/group).

Parameters	Groups			
	Control	SP	LG	LG + SP
Initial body weight (g/fish)	49.80 ± 0.49	50.20 ± 0.37	49.6 ± 0.51	49.4 ± 0.68
Final body weight (g/fish)	87.60 ± 0.81^c^	98.80 ± 0.80^a^	95.6 ± 0.51^b^	94.6 ± 0.51^b^
Weight gain (g/fish)	37.80 ± 0.66^c^	48.60 ± 0.68^a^	46.0 ± 0.32^b^	45.20 ± 0.20^b^
Feed intake (g/fish/2 months)	98.85 ± 0.52	97.14 ± 2.06	98.96 ± 1.45	99.96 ± 1.51
FCR	2.62 ± 0.04^a^	2.0 ± 0.03^c^	2.15 ± 0.02^b^	2.21 ± 0.04^b^

Values are expressed as mean ± standard errors. Means in the same row (a-c) with different letters significantly differ at (*P* ≤ 0.05).

*FCR* feed conversion ratio.

### Blood and immunological assays

The leverage of lemongrass and/or *A. platensis* on the blood and immunological assays of Nile tilapia are shown in Tables 5 and 6 before and after experimental infection. Before infection, the statistical analysis of the collected data on the RBCs, Hb, and PCV significantly (*P* < 0.05) improved in the all-treatment groups when compared with the control group. These improvements were superior to those in the LG + SP group. After infection, the RBCs of the LG + SP group were not affected by the experimental infection. Meanwhile, PCV and Hb significantly (*P* < 0.05) decreased in the LG + SP group when compared with the same group before infection. On the other side, the data of the hematological parameters of the *A. platensis* and LG groups significantly decreased in contrast to the same groups prior to infection (Table 5).

**Table 5:** Impact of *A. platensis* and Lemongrass on nile tilapia hematological parameters experimentally infected with *A. hydrophila* (*n* = 9).

Parameters	Groups				
	Time	Control	SP	LG	LG + SP
RBCs count (× 106/µl)	Before infection	1.68 ± 0.04^cX^	1.86 ± 0.04^bX^	2.16 ± 0.26^abX^	2.45 ± 0.04 ^aX^
	After infection	1.41 ± 0.05^cY^	1.70 ± 0.04^bcY^	1.86 ± 0.16^bX^	2.34 ± 0.07^aX^
Hb (g/dl)	Before infection	6.47 ± 0.34^cX^	7.95 ± 0.22^bX^	8.14 ± 0.41^bX^	9.41 ± 0.25^aX^
	After infection	4.00 ± 0.23^bY^	6.43 ± 0.38^aY^	6.40 ± 0.29^aY^	7.27 ± 0.46^aY^
PCV%	Before infection	18.55 ± 0.40^cX^	23.50 ± 0.73^bX^	24.13 ± 0.23^bX^	28.36 ± 0.34^aX^
	After infection	10.34 ± 0.77^cY^	18.44 ± 0.11^bY^	18.33 ± 0.96^bY^	21.22 ± 0.40^aY^

Values are expressed as mean ± standard errors. Means in the same row (a–c) with different letters significantly differ at (*P* ≤ 0.05) meanwhile means in the same column (X–Y) with different letters significantly differ at (*P* ≤ 0.05).

**Table 6:** Impact of *A. platensis* and Lemongrass on nile tilapia differential leukocytic count experimentally infected with *A. hydrophila* (*n* = 9).

Parameters	Groups				
	Time	Control	SP	LG	LG + SP
WBCs count (× 10^3^/µl)	Before infection	20.9 ± 0.2^Y^	20.27 ± 0.1^Y^	20.03 ± 0.05^Y^	20.48 ± 0.51 ^Y^
	After infection	43.53 ± 1.04^aX^	28.6 ± 1.2^bcX^	31.79 ± 0.92^bX^	27 ± 0.92^cX^
Heterophils (× 10^3^/µl)	Before infection	6.9 ± 0.4^aY^	6.6 ± 0.2^abY^	6.11 ± 0.49^abY^	5.42 ± 0.33^bY^
	After infection	24.15 ± 0.85^aX^	11.5 ± 0.7^bX^	13.63 ± 0.62^bX^	6.95 ± 0.58^cX^
Lymphocyte (× 10^3^/µl)	Before infection	8.2 ± 0.3^cX^	9.1 ± 0.1^bX^	8.93 ± 0.19^bX^	11.27 ± 0.04^ay^
	After infection	6.4 ± 0.26^cY^	9.3 ± 0.5^bX^	9.9 ± 0.15^bX^	14.38 ± 0.27^aX^
Monocytes (× 10^3^/µl)	Before infection	5.1 ± 0.0^aY^	4.1 ± 0.0^bY^	4.46 ± 0.22^bY^	3.34 ± 0.11^cY^
	After infection	12.16 ± 0.46^aX^	7.1 ± 0.04^bX^	7.56 ± 0.17^bX^	5.05 ± 0.06^cX^
Eosinophiles (× 10^3^/µl)	Before infection	0.60 ± 0.0^aY^	0.54 ± 0.0^bY^	0.55 ± 0.01^bY^	0.46 ± 0.03^cY^
	After infection	0.83 ± 0.01^aX^	0.73 ± 0.01^bX^	0.71 ± 0.02^bX^	0.62 ± 0.01^cX^

Values are expressed as mean ± standard errors. Means in the same row (a–c) with different letters significantly differ at (*P* ≤ 0.05) meanwhile means in the same column (X–Y) with different letters significantly differ at (*P* ≤ 0.05).

However, the immunological assay’s findings demonstrated that the WBC count in each group before infection was unaffected. Following infection, the three treatment groups’ counts were lower than those of the control group. The lymphocyte count was higher in the three treatment groups prior to and following infection in contrast to the control group. Prior to and following infection, the three treatment groups’ counts of basophils, monocytes, and heterophils were lower than those of the control group (Table 6).

### Serum parameters

Before and after infection, the liver enzymes (ALT, AST, and alkaline phosphatase) were considerably (*P* < 0.05) lower in the all-treatment groups than in the control group. This decline was better in the LG + SP group. When compared to the same group prior to infection, the control group’s liver enzyme activities significantly increased after infection (Table 7).

**Table 7:** Impact of *A. platensis* and Lemongrass on nile tilapia liver enzymes parameters experimentally infected with *A. hydrophila* (*n* = 9).

Parameters	Groups				
	Time	Control	SP	LG	LG + SP
AST (U/L)	Before infection	48.13 ± 0.17^ay^	47.36 ± 0.16^aby^	46.65 ± 0.84^aby^	46.08 ± 0.35^by^
	After infection	110.68 ± 0.75^ax^	90.90 ± 0.80^bx^	88.87 ± 0.90^bx^	79.10 ± 0.42^cx^
ALT (U/L)	Before infection	13.22 ± 0.34^ay^	12.54 ± 0.12^aby^	12.45 ± 0.18^by^	12.22 ± 0.12^by^
	After infection	33.62 ± 0.53^ax^	19.6 ± 0.44^bx^	19.22 ± 0.62^bcx^	17.88 ± 0.25^cx^
ALP (U/L)	Before infection	34.48 ± 0.70^ay^	32.45 ± 0.42^by^	28.88 ± 0.68^cy^	20.92 ± 0.10^dy^
	After infection	56.4 ± 0.75^ax^	44.75 ± 0.89^bx^	43.85 ± 0.26^bcx^	42.42 ± 0.11^cx^

Values are expressed as mean ± standard errors. Means in the same row (a–d) with different letters significantly differ at (*P* ≤ 0.05) meanwhile means in the same column (X–Y) with different letters significantly differ at (*P* ≤ 0.05).

Regarding the results of the serum proteins and kidney function, the combination treatment (LG + SP) higher serum total protein, albumin, and globulin levels before infection. Meanwhile, creatinine and urea decreased in the same group when compared with the other groups before the infection. After infection, compared to the same group prior to infection, all groups’ blood protein and kidney function test levels dropped, except albumin in the SP group (Table 8).

**Table 8:** Impact of *A. platensis* and Lemongrass on nile tilapia liver and kidney functions experimentally infected with *A. hydrophila* (*n* = 9).

Parameters	Groups				
	Time	Control	SP	LG	LG + SP
Total protein	Before infection	4.9 ± 0.40^bX^	4.9 ± 0.15^Bx^	4.93 ± 0.09^bX^	6 ± 0.53^aX^
	After infection	3.71 ± 0.05^bY^	4.28 ± 0.12^aY^	4.44 ± 0.12^aY^	4.75 ± 0.23^aY^
Albumin	Before infection	1.3 ± 0.06^bX^	1.33 ± 0.03^bX^	1.3 ± 0.058^bX^	1.6 ± 0.06^aX^
	After infection	0.93 ± 0.02^bY^	1.4 ± 0.09^aX^	1.15 ± 0.07^abY^	1.31 ± 0.13^aY^
Globulin	Before infection	3.6 ± 0.36^bX^	3.57 ± 0.12^abX^	3.63 ± 0.13^abX^	4.4 ± 0.51^aX^
	After infection	2.78 ± 0.07^Y^	2.88 ± 0.10^Y^	3.29 ± 0.13^Y^	3.44 ± 0.32^Y^
Urea (mg/L)	Before infection	7.9 ± 0.40^aY^	6.73 ± 0.32^bY^	5.93 ± 0.33^bY^	4.83 ± 0.22^cY^
	After infection	12.06 ± 0.31^aX^	9.26 ± 0.39^bX^	11 ± 0.27^aX^	8.74 ± 0.58^bX^
Creatinine (mg/L)	Before infection	1.23 ± 0.03^aY^	1.03 ± 0.07^bcY^	1.07 ± 0.03^bY^	0.9 ± 0.06^cY^
	After infection	1.63 ± 0.07^aX^	1.3 ± 0.06^bX^	1.32 ± 0.06^bX^	1.27 ± 0.03^bX^

Values are expressed as mean ± standard errors. Means in the same row (a–c) with different letters significantly differ at (*P* ≤ 0.05) meanwhile means in the same column (X–Y) with different letters significantly differ at (*P* ≤ 0.05).

### Phagocytic activity and index

The leverage of lemongrass and/or *A. platensis* on the phagocytic activity and index of Nile tilapia are shown in Table 9 before and after the experimental infection. The results of the three treatment groups were significantly (*P* < 0.05) higher than the results of the control group, with a superior effect to the LG + SP group.

**Table 9:** Impact of *A. platensis* and Lemongrass on nile tilapia phagocytic activity and index experimentally infected with *A. hydrophila* (*n* = 9).

Parameters	Groups				
	Time	Control	SP	LG	LG + SP
Phagocytic activity %	Before infection	21.1 ± 0.3^cX^	25.8 ± 0.8^bX^	24.9 ± 0.3^bX^	31.6 ± 0.6^aX^
	After infection	15.0 ± 0.2^dY^	22.3 ± 0.8^bY^	19.9 ± 0.2^cY^	26.4 ± 0.4^aY^
Phagocytic index %	Before infection	2.3 ± 0.1^cX^	3.0 ± 0.1^bX^	3.0 ± 0.0^bX^	4.2 ± 0.1^aX^
	After infection	1.2 ± 0.0^cY^	2.4 ± 0.1^by^	2.2 ± 0.0^bY^	3.0 ± 0.0^aY^

Values are expressed as mean ± standard errors. Means in the same row (a–c) with different letters significantly differ at (*P* ≤ 0.05) meanwhile means in the same column (X–Y) with different letters significantly differ at (*P* ≤ 0.05).

### Histopathological finding and intestinal morphometry

**Fig. 2:**
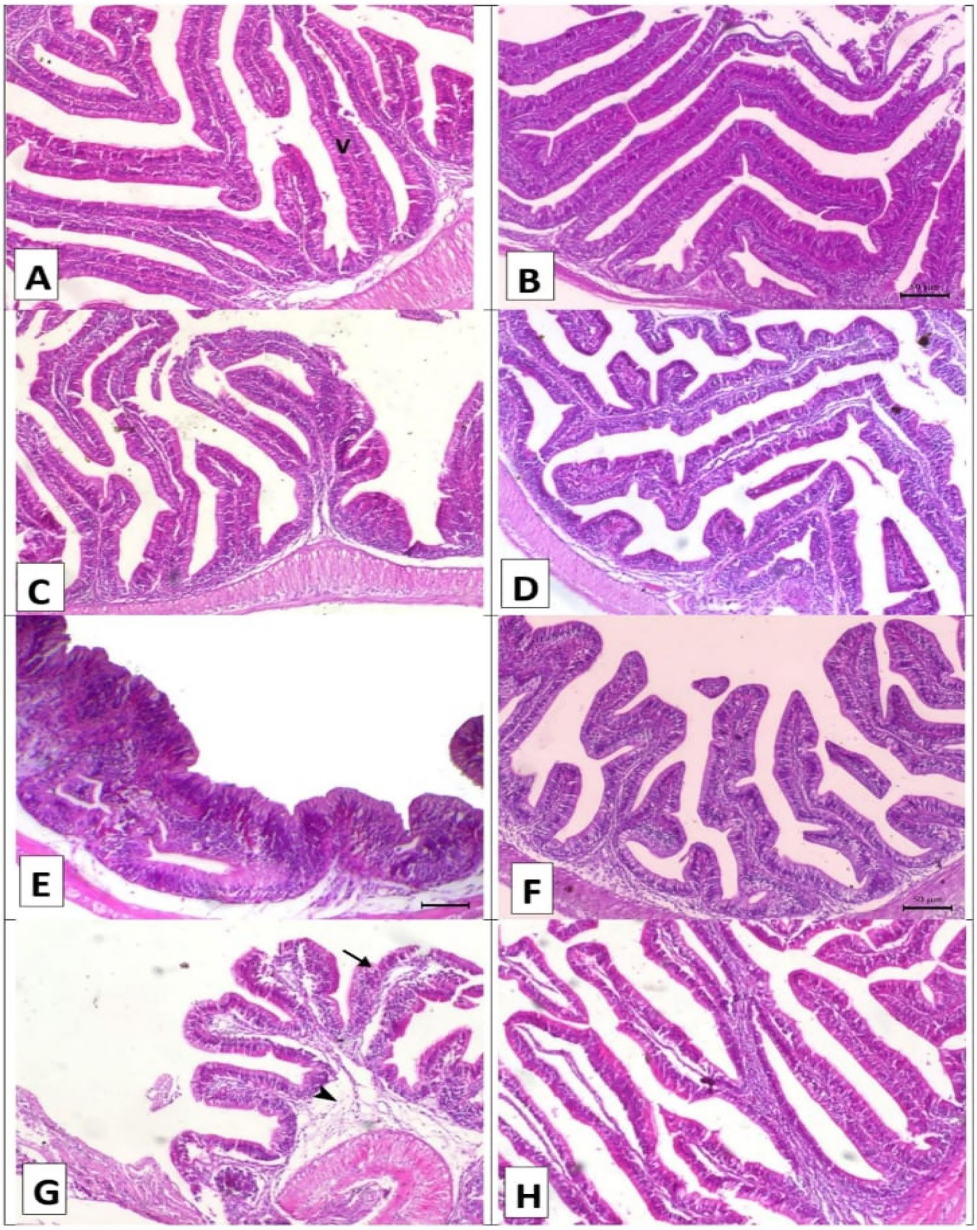
Displayed intestinal histopathology stained with H&E prior to and after artificial infection. (**A–D**) for the control, SP, LG, and LG + SP non infected groups showed normal intestine histology with normal villous (V). (**E**) The control infected group showed diffuse villous necrosis (arrow) with marked submucosal edema and inflammatory cells infiltration (arrowhead). (**F**) The *A. platensis* infected group showed normal necrosis of individual villous (arrow) with mild submucosal edema (arrowhead). (**G**) The LG infected group showed necrosis of some villi (arrow) with significant submucosal edema (arrowhead), and (**H**) the LG + SP infected group showed normal intestine histology. x 160.

**Fig. 3:**
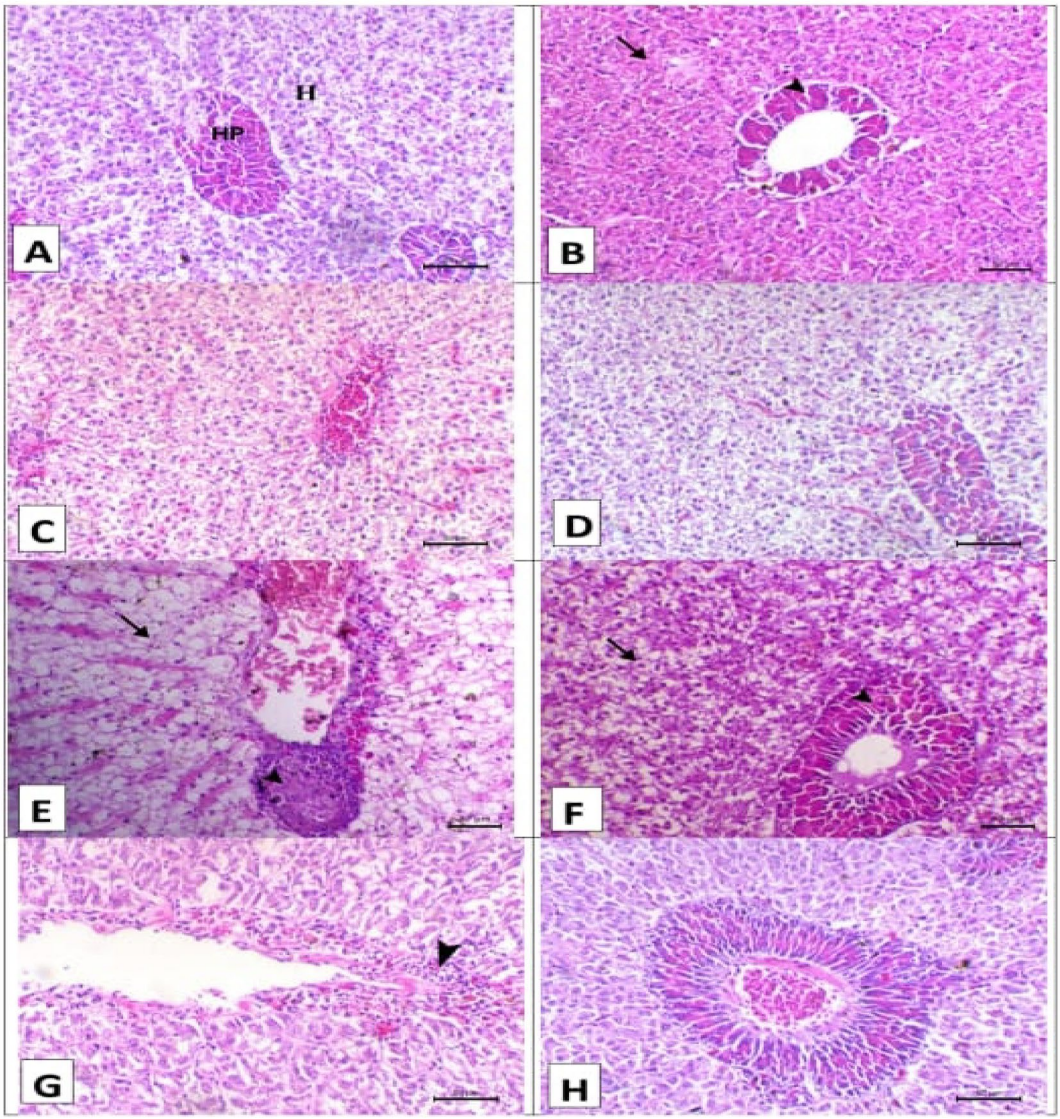
Displayed hepatopancreas histopathology stained with H&E prior to and after artificial infection. (**A–D**) for the control, SP, LG, and LG + SP non infected groups showed normal hepatopancreatic histology where hepatocytes cords (H) and pancreatic acini surrounding central viens (HP). (**E**) The control infected group showed widespread hepatopancreatic necrosis. (**F**) The *A. platensis* group infected with *Aeromonas* shown hepatic necrosis. (**G**) The LG infected group showed pancreatic necrosis (arrowhead), and (**H**) the LG + SP infected group showed normal hepatopancreatic histology. Scale bar = 50 μm.

**Fig. 4:**
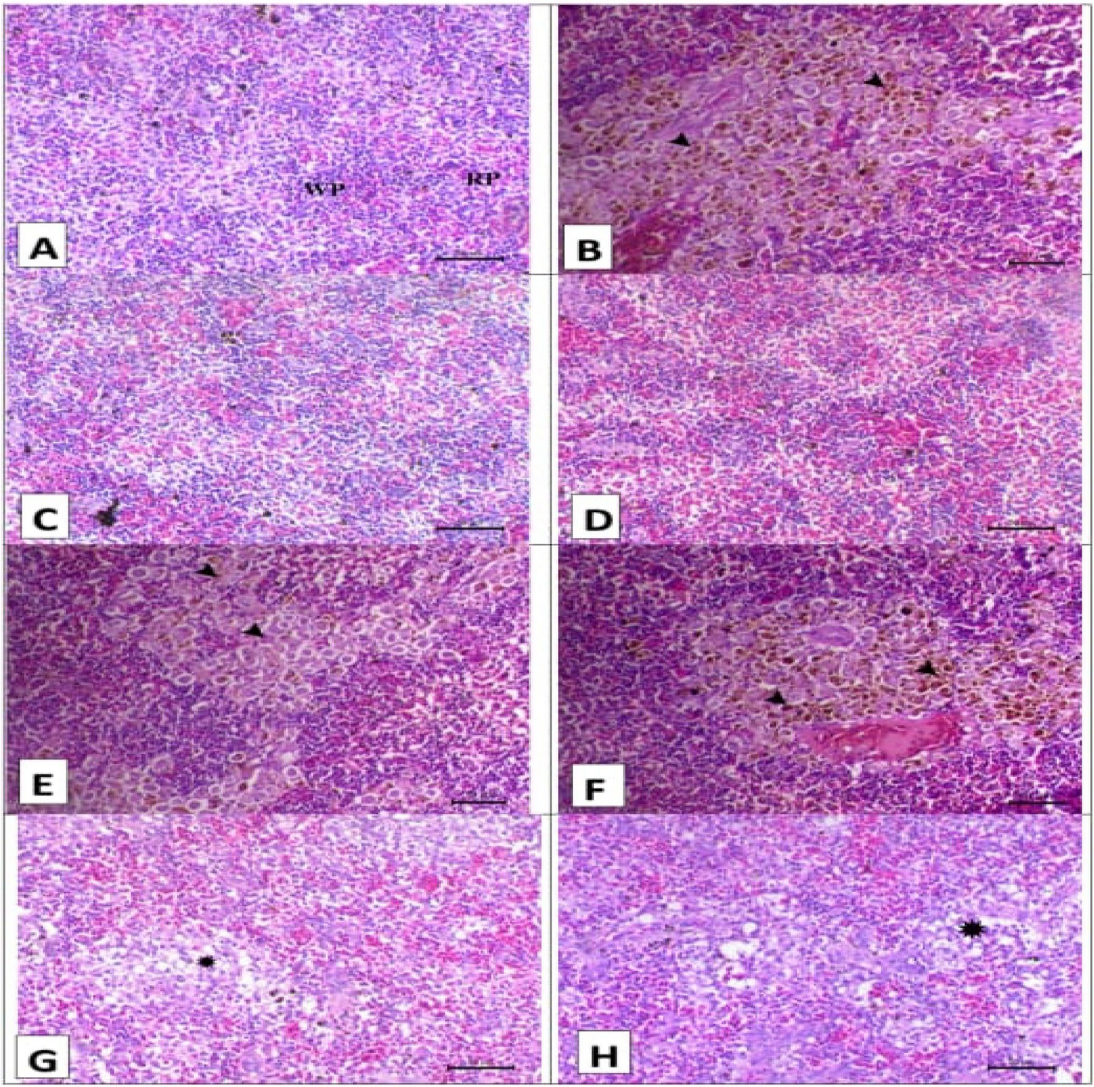
Displayed spleen histopathology stained with H&E prior to and after artificial infection. (**A–D**) for the control, SP, LG, and LG + SP non infected groups showed normal histology consisted mainly of white pulp that mainly contains lymphocytes (WP) and red pulp that mainly contains erythrocytes (RP). (**E**) The control infected group showed marked depletion of white pulps (asterisks) and activation of melanomacrophage center (arrowhead). (**F**) The *A. platensis* infected group showed moderate depletion of white pulps (asterisk) and activation of melanomacrophage center (arrowhead). (**G**) The LG infected group showed moderate depletion of white pulps (asterisk), and (**H**) the LG + SP infected group showed mild depletion of white pulp (asterisk). Scale bar = 50 μm.

**Fig. 5:**
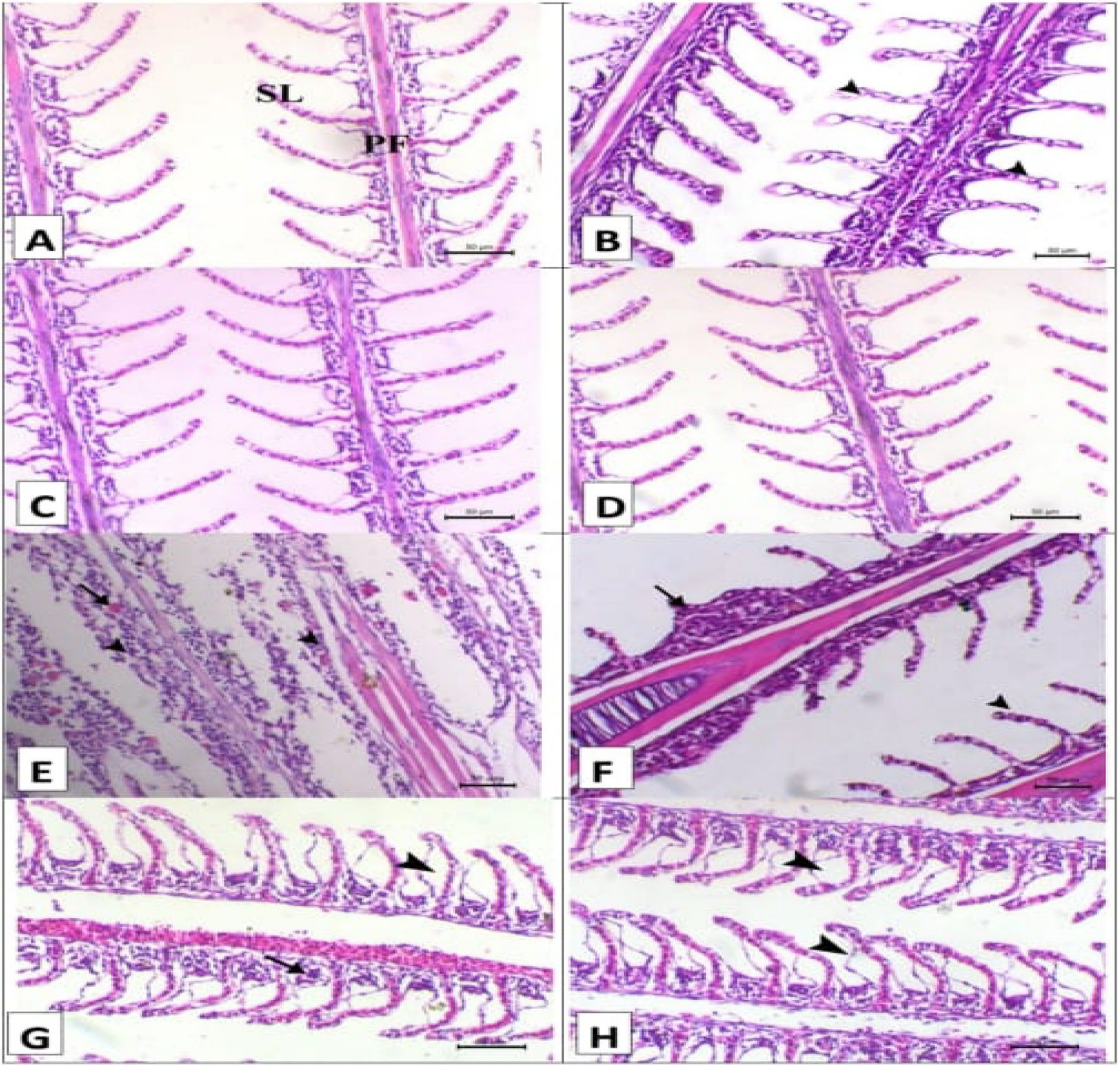
Displayed gills histopathology stained with H&E prior to and after artificial infection. (**A–D**) for the control, SP, LG, and LG + SP non infected groups showed normal histology consisted mainly of primary filament (PF) branch out into tiny secondary lamellae (SL). (**E**) The control infected group showed widespread diffuse filamentous epithelial necrosis (arrowheads). (**F**) The *A. platensis* infected group showed multifocal filamentous epithelial necrosis (arrowheads). (**G**) The LG infected group showed focal filamentous epithelial necrosis (arrow) and diffuse lifting of the covering epithilium of the secondary lamellae (arrowhead), and (**H**) The LG + SP infected group showed diffuse lifting of the covering epithilium of the secondary lamellae (arrowheads). Scale bar = 50 μm.

Figures 2, 3 and 4, and 5 shows the impact of *A. platensis* and lemongrass oil on the intestine, liver, spleen, and gills pathology. Before each group was experimentally infected, the intestinal photomicrograph; (Fig. 2A–D) for the control, SP, LG, and LG + SP groups, respectively, showed normal intestine histo-architecture with normal villous. After experimental infection, there was noticeable submucosal edema, inflammatory cell infiltration, and diffuse villous necrosis in the control infected group (Fig. 2E). Individual villous necrosis was normal in the SP-infected group, and there was modest submucosal edema (Fig. 2F). Significant submucosal edema and necrosis of several villi were observed in the LG-infected group (Fig. 2G). The intestinal histoarchitecture was normal in the LG + SP-infected group (Fig. 2H).

About the hepatopancreas photomicrographs of the various groups prior to experimental infection; (Fig. 3A–D): Normal hepatopancreatic histo-architecture was observed in the control, SP, LG, and LG + SP groups, respectively, with hepatocyte cords and pancreatic acini encircling the central viens. The control group experienced extensive hepatopancreatic necrosis following the experimental infection (Fig. 3E). The SP group showed hepatic necrosis (Fig. 3F) after infection. The LG group showed pancreatic necrosis (Fig. 3G) after infection. There was normal hepatopancreatic histoarchitecture in the LG + SP group (Fig. 3H) after infection.

The spleen photomicrographs of the various groups prior to experimental infection are shown in Fig. 4A–D. Normal histoarchitecture was displayed by the control, SP, LG, and LG + SP groups, respectively. This included red pulp that primarily contained erythrocytes and white pulp that primarily included lymphocytes. The control group displayed significant white pulp loss and melanomacrophage center activation upon infection (Fig. 4E). The melanomacrophage center was activated and white pulps were moderately depleted in the SP group upon infection (Fig. 4F). White pulp depletion was moderate in the LG group upon infection (Fig. 4G). The LG + SP group showed mild depletion of white pulp (Fig. 4H).

The gills photomicrographs of the various groups prior to experimental infection; (Fig. 5A–D): the control, SP, LG, and LG + SP groups, respectively, showed normal histo-architecture consisting mainly of primary filament branching out into tiny secondary lamellae. The control group displayed extensive diffuse filamentous epithelial necrosis following infection (Fig. 5E). Multifocal filamentous epithelial necrosis was seen in the SP group following infection (Fig. 5F). The LG group displayed diffuse lifting of the secondary lamellae’s covering epithelium and localized filamentous epithelial necrosis following infection (Fig. 5G). The secondary lamellae’s covering epithelium was diffusely lifted in the LG + SP group following infection (Fig. 5H).

In terms of intestinal morphometry, the SP, LG, and LG + SP groups outperformed the control group in terms of villi length and width both before and after infection. Meanwhile, inter villi space was higher in the control group when compared with the SP, LG, and LG + SP groups (Table 10).

**Table 10:** Impact of *A. platensis* and Lemongrass on nile tilapia intestinal morphometry experimentally infected with *A. hydrophila* (*n* = 5).

Parameters	Groups				
	Time	Control	SP	LG	LG + SP group
Villi length (um)	Before	447 ± 1.6^dX^	524 ± 1.8^bX^	496.9 ± 1.9^cX^	621.5 ± 0.9^aX^
	After	203.5 ± 1.1^dY^	375 ± 1.0^bY^	349.9 ± 0.6^cY^	461.7 ± 1.1^aY^
Villi width (um)	Before	58.1 ± 1.2^dX^	60 ± 0.4^cX^	74.1 ± 3.4^bX^	105.5 ± 1.7^aX^
	After	52.5 ± 1.75^cY^	56 ± 0.2^bY^	55 ± 1.8^bY^	83.3 ± 1.8^aY^
Inter villi space (um)	Before	68 ± 1.5^aY^	42 ± 0.6^cY^	57.4 ± 1.7^bY^	35.6 ± 0.8^dY^
	After	104.1 ± 0.7^aX^	83.4 ± 0.5^bX^	79.7 ± 2.6^dX^	60.5 ± 0.6^cX^

Values are expressed as mean ± standard errors. Means in the same row (a–c) with different letters significantly differ at (*P* ≤ 0.05) mean while means in the same column (X–Y) with different letters significantly differ at (*P* ≤ 0.05).

### Gene expression

**Fig. 6:**
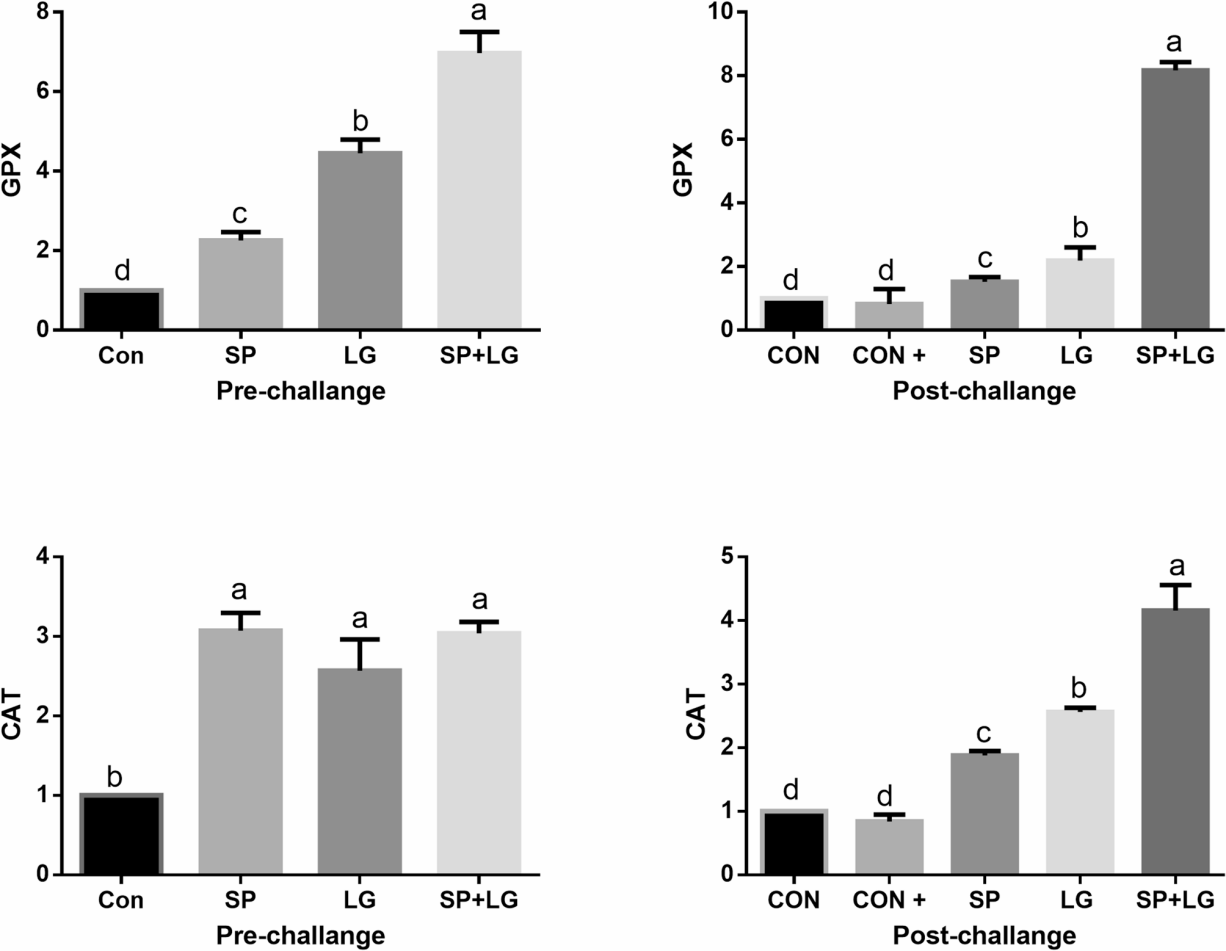
Impact of *A. platensis* and lemongrass in the diet of Nile tilapia on liver *GPX* and *CAT* mRNA transcript level prior to and after artificial infection. Values were expressed as Mean ± SE. Columns with different litters indicates statistically significant values with *P-values* ≤ 0.05.

Significant interactive effects of dietary *A. platensis* and lemongrass inclusion were observed on the expression of the antioxidant-related genes (*gpx* and *cat*) in the liver tissue of *O. niloticus* prior to and upon infection, as shown in Figure 6. The inclusion of *A. platensis* and lemongrass alone or in combination in the diet of fish significantly (*P* ≤ 0.05) upregulated the *gpx* and *cat* expression with superior effects to combination inclusion. However, the bacterial infection alone was associated with a slight down regulation of both *cat* and *gpx* expression compared to the control non infected group (*P* ≤ 0.05).

**Fig. 7:**
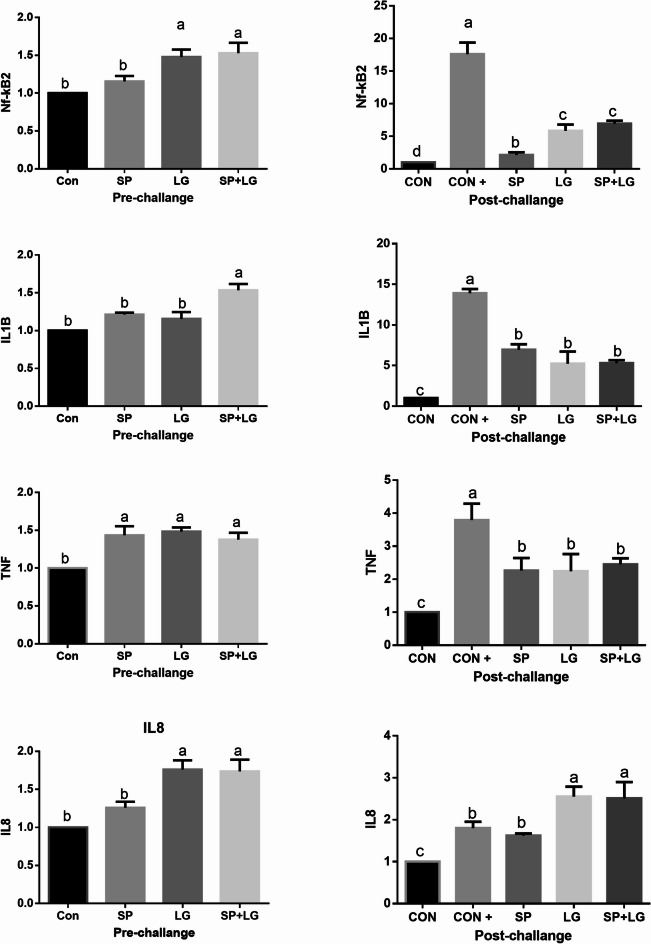
Impact of *A. platensis* and lemongrass in the diet of Nile tilapia on liver *NKB*,* IL1B*,* TNF*, and *IL8* mRNA transcript level prior to and after artificial infection. Values were expressed as Mean ± SE. Columns with different litters indicates statistically significant values with *P-values* ≤ 0.05.

Moreover, there were significant interactive effects of dietary *A. platensis* and lemongrass inclusion on the expression of proinflammatory-related genes (*nfkb2*,* il1β*,* tnf*, and *il8)* in the liver tissue of *O. niloticus* prior to and upon infection, as shown in Figure 7. The inclusion of lemongrass alone or in combination with *A. platensis* in the diet of fish significantly (*P* ≤ 0.05) upregulated *nlb* and *ilβ* expression. Combination treatment increased the mRNA transcription levels of *il1β*. Meanwhile, *tnf* was upregulated in the tree treatment groups. On the other hand, infection increased the *mRNA* transcription levels of *nfkb2*,* il1β*, and *tnf*. Meanwhile, infection downregulated *il8*.

## Discussion

In aquaculture, the use of phytogenic compounds in fish diets has recently gained a lot of attention^33^. The results of Okasha et al.^34^ and Metekia and Ulusoy^35^ on *A. platensis* in the *O. niloticus* against experimental infection are combatable with the results of this trial. Additionally, Mahmoud et al.^36^ observed supplementation with *A. platensis* (1%) improved against *Pseudomonas fluorescence* in Nile tilapia. This effect may be attributed to the carotenoids that are comprised in the *A. platensis*, which reduce stress and bolster the body’s defenses against infection^37^. Moreover, Abdo et al.^38^ demonstrated that bioactive substances found in *A. platensis* include antibacterial, anti-inflammatory, and antioxidant qualities. On the other hand, the outcomes of the clinical manifestations of the LG group after experimental infection are in harmony with Sukrakanchana et al.^39^, who found that lemongrass oil decreased mortality in Nile tilapia after *Streptococcus agalactiae* infection. Moreover, Al-Sagheer et al.^27^ observed resistance of the diseases by lemongrass oil in the Nile tilapia and ascribed this resistance to the growth promoting effect of lemongrass. There may be a clear correlation between the potent antibacterial properties of lemongrass and their growth-promoting effects^40^. On the other hand, Oussalah et al.^41^ attributed this effect to their active constituents, which include 1,8-cineol, α-pinene, and limonene. However, this positive effect was pronounced in the LG + SP group. Similar finding was observed by Abdo et al.^38^, who reported that remarkably, the Nile tilapias fed with LEO and *A. platensis* showed the highest immune response, suggesting that their actions were synergistic. Mohammed et al.^42^ reported the immunomodulatory effect of combination treatment with rosemary and *A. platensis* in Nile tilapia. Meanwhile, Copatti et al.^9^ found that lemongrass did not affect the survival of tambaqui after experimental infection.‏ On the other hand, Fadl et al.^43^ and Yardimci and Aydin^44^ reported similar results to those of the control group in Nile tilapia after experimental infection with *A. hydrophila.*

In the current study, the addition of *A. platensis* and lemongrass to the Nile tilapia fish feed improved growth parameters. Similar findings were obtained by Al-Deriny et al.^45^ and Al-Sagheer et al.^27^ in *O. niloticus* with *A. platensis* and lemongrass, respectively. The growth parameters include, body weight and gain, specific growth rate, and FCR of *O. niloticus* improved by dietary supplementation with *A. platensis*^46^. Moreover, Amer^47^ reported that *A. platensis* had a higher body weight and a lower FCR with Nile tilapia. Similar results were obtained by El-Araby et al.^48^. These results are confirmed by the results of the intestinal morphometry. By enhancing intestinal morphometry and stimulating the GH/IGF axis, *A. platensis* has a favorable impact on *O. niloticus* growth^49^. Moreover, Sherif et al.^50^ found that *A. platensis* increased activity of the Nile tilapia intestinal enzymes. On the other hand, Adeniyi^51^ reported that lemongrass inclusion in the diet of *Clarias gariepinus* increased body weight and gain and lowered FCR. Similar findings were reported by Gichana^52^ in Nile tilapia. Moreover, Orzuna-Orzuna and Granados-Rivera^53^ reported that *O. niloticus* growth, antioxidant in the serum, and intestinal morphometry can all be enhanced by adding essential oils to their diet. On the contrary, Mahmoud et al.^36^ found that supplementation with 1% *A. platensis* did not improve the growth of Nile tilapia. Also, inclusions of lemongrass leaf at higher levels decreased body weight and weight gain and increased the FCR of African catfish^54^. This may be attributed to the anti-nutritional factors in lemongrass leaves^55^. This difference could be attributed to the status of the lemongrass used (leaf or oil) and the species of fish used in the experiment. However, the combination treatment (LG + SP) significantly affected the growth of *O. niloticus*. Similar finding was observed by Abdo et al.^38^, who reported that remarkably, the Nile tilapias fed with LEO and *A. platensis* showed the highest growth performance, suggesting that their actions were synergistic. Mohammed et al.^42^ reported the growth promoting effect of combination treatment with rosemary and *A. platensis* in Nile tilapia.

Regarding the results of the hematology and immune response before infection, these results similar to Mahmoud et al.^36^, who found that supplementation with 1% *A. platensis* improved hematological parameters and antioxidant enzymes in the serum of Nile tilapia. Moreover, Abdel-Tawwab et al.^56^ reported that *A. platensis* improved the growth and hemato-biochemical parameters of the *O. niloticus*. 7.5 g *A. platensis*/kg diet significantly increased RBCs, PCV, and hemoglobulin in Nile tilapia fish^57^. Moreover, Youssef et al.^46^ and El-Sheekh et al.^58^ reported that *A. platensis* inclusion increased the RBCs and haemoglobin of Nile tilapia and hybrid red tilapia, respectively. Similar results were obtained by Sarma et al.^59^, who attributed this increase to the hemopiotic stimulation effect of *A. platensis*. On the other hand, Thuong et al.^60^ found that lemongrass oil positively affected the haematology of the red tilapia. However, these results are confirmed by the results of Mohammady et al.^61^, who reported the positive effect of the phytogenic extract on the hematology of the *O. niloticus*. On the contrary, Al-Zayat^57^ reported that *A. platensis* inclusion increased WBCs. Sayed and Fawzy^62^ and Youssef et al.^46^ have observed comparable outcomes in catfish and *O. niloticus*, respectively. Lemongrass oil positively affected the WBCs of the red tilapia^60^. The fish type and the source of the lemongrass and *A. platensis* utilized in each study could be the cause of this discrepancy.

Upon infection, the findings of the haematological and immunological parameters were similar to the findings of Fadl et al.^43^, who reported decreased and increased haematological and immunological parameters in the control group, respectively. Similar findings were reported by Talpur et al.^63^, Hardi et al.^64^, and Ergena et al.^65^ in Nile tilapia experimentally infected with *A. hydrophila*. However, reduced red blood cell numbers signify that the infection is affecting or destroying erythrocytes. Fish that are diseased as a result of infection are likely to exhibit changes in their haematology^66^. These negative effects were ameliorated by *A. platensis* and lemongrass inclusion. Similar results were obtained by Fadl et al.^43^ with microalgae and *A. hydrophila*. *A. platensis* has a beneficial effect on the antibacterial activity against *A. hydrophila* in *O. niloticus*^67^. Moreover, Thuong et al.^60^ found that lemongrass improved hematological parameters of red tilapia after experimental infection with *Streptococcus agalactiae*.‏

Liver enzymes such (ALT and AST) are important indicators of liver function. Increased serum activities of liver enzymes indicate liver damage^68^. The results of the serum liver enzymes in the present trial before infection decreased with different treatments, especially the combination treatment. Similar findings were obtained by Fadl et al.^43^ with *A. platensis* and Li et al.^69^ with lemongrass oil. Meanwhile, Abo El-Ward et al.^70^ found opposite results. Additionally, *A. platensis* addition had no influence on serum liver enzyme activity^46^. However, Gamboa-Delgado et al.^71^ reported the hepatoprotective effect of *A. platensis* in *O. niloticus*.

On the other side, Souza et al.^72^ reported that lemongrass oil increased serum ALT and AST in Nile tilapia. Following infection, the control infected group’s liver enzyme activity rose. The findings of Fadl et al.^43^ support these findings. Moreover, Bekele et al.^73^ and Martins et al.^74^ reported that liver enzymes increased after experimental infection with vibrio bacteria and *Enterococcus*, respectively. Meanwhile, the outcomes of *A. platensis* after infection align with the findings of Fadl et al.^43^, who found that feeding Nile tilapia *A. platensis* reduced their liver enzyme activity. To the best of our knowledge, no studies have been conducted on the measures of the liver enzymes that activate in fish that are fed lemongrass oil following an experimental infection.

Dietary inclusion with a combination of lemongrass oil and *A. platensis* has shown controversial results on *O. niloticus* serum markers. Serum proteins in the LG + SP group showed significant increases, while serum kidney function tests decreased in all treatment groups when compared with the control group before infection. Acar et al.^75^ and Baba et al.^76^ found that diets that include sweet orange and lemon peel essential oils at concentrations of 3 to 80 g/kg of feed have been shown to raise plasma protein levels of *O. niloticus*. Additionally, Mohammed et al.^42^ highlighted the beneficial impact of combination treatment with rosemary and *A. platensis* on serum proteins of Nile tilapia. Meanwhile, Saccol et al.^77^ discovered that the serum proteins, urea, and creatinine of silver catfish were not affected by lemongrass oil. Similar results were obtained by Rampelotto et al.^78^ in silver catfish. However, Al-Zayat^57^ found that a 7.5 g *A. platensis* /kg diet significantly improved serum total protein and globulin and decreased levels of albumin, urea, and creatinine and the activities of liver enzymes. Meanwhile, Youssef et al.^46^ reported that dietary inclusion of *A. platensis* had no effect on the A/G ratio, globulin levels, or blood total protein but decreased urea and creatinine. Also, Ref^45^. found that *A. platensis* supplementation does not affect serum proteins. After infection, serum proteins and urea and creatinine decreased and increased, respectively, in contrast to the same group prior to infection. Fadl et al. achieved similar outcomes^43^.

However, the phagocytic index and activity results, which improved with various treatments both prior to and upon the experimental infection, supported the serum protein results. Similar findings were observed by Mahmoud et al.^36^, who found that supplementation with *A. platensis* (1%) increased the bactericidal, phagocytic, and lysozyme activities of Nile tilapia. *A. platensis* increased phagocytic and lysosomal activity in the blood of Nile tilapia^46^. Lemongrass oil increased the phagocytic index and activity of broilers^79^. Lemongrass oil increased the lysosomal activity of *O. niloticus*^27^. Similar results were reported by Dawood et al.^80^ and Sutili et al.^81^ with essential oil extract and citral in Nile tilapia and silver catfish, respectively. In channel catfish, feeding 0.02–0.05% of essential oils from oregano, lemongrass, and geranium boosted lysozyme and catalase activity^82^.

Inclusion of *A. platensis* and lemongrass oil did not affect the histopathology of different organs before infection, and a reduced pathological lesion resulted from the experimental infection. Similar findings were cited by Abdo et al.^38^, who reported that tissue histology of the Nile tilapias fed with LEO and *A. platensis* does not affect. Also, Mohammed et al.^42^ highlighted the beneficial impact of combination treatment with rosemary and *A. platensis* in Nile tilapia on organs histopathology. Awe et al.^83^, who reported that lemongrass oil did not impact the liver and gills histology of African mud catfish upon experimental infection with *Aeromonas veronii*. Also, Dobhal et al.^84^ highlighted the beneficial impact of lemongrass oil on the liver and pancreas of rats with metabolic syndrome. These results may be attributed to the anti-inflammatory effect of lemongrass oil^85^ or to its component polyphenols, which have hepatoprotective effects^84^. Moreover, Huyben et al.^86^ reported that plant essential oils increase the villi length of rainbow trout. *Arthrospira platensis* inclusion increased intestinal villi height and width and counts of lymphocytes and goblet cells in the intestine of Nile tilapia^46^.

However, all the above-mentioned results were confirmed by the results of gene expression before and after infection. The findings of the antioxidant genes are consistent with the results of Mohammady et al.^61^, who found that a phytogenic mixture improves the expression of the antioxidant-related genes in Nile tilapia. Similar findings were obtained by Teimouri et al.^87^ with *A. platensis* in rainbow trout. In contrast, according to research by Chekani et al.^88^, the expression of immune-associated genes has significantly improved without effect on the antioxidant genes while using dietary herbal feed supplements in *O. mykiss*.

Proinflammatory cytokine genes were chosen because of their significance as indicators of inflammation and stress^89^. Monitoring cytokines such as *il-1β* is important for the immune response alteration^90,91^. In the current study, the upregulation (about 1.5:2-fold change) of the genes *il-1β*,* il8*, and *tnf*-α in the non-infected group could refer to an active immune system. The *il1* family has an important role in the innate immune response under normal conditions^92,93^. In accordance, it was reported that dietary feed additives such as ferulic acid and *Pediocuccus pentosaceus* significantly increased the *mRNA* levels of *il-1β*,* il8*, and *tnf-α* in rainbow trout cultured under normal conditions^94^. A current investigation supports the notion that, *A. platensis* causes the *tnf-α* gene in carp^95^.

The enhanced expression of proinflammatory cytokines is essential to overcome infection and oxidative stress conditions. The supplementation of *A. platensis* and/or lemongrass modulated the cytokine expression after the bacterial infection, as the relative expressions of *nfkb2*,* il-1β*, and *tnf-α* were lower than the control infected group. Moreover, SP and/or lemon supplementation significantly enhanced antioxidant genes expression and alleviated tissue necrosis signs in the different organs after infection. In line with our results, *A. platensis* enhanced *tnf-α* gene expression in Nile tilapia^36^. When *A. platensis* was added to the diet of rainbow trout, the expression of the *tnf*-α gene increased^96^. Moreover, Hassaan et al.^97^ reported the upregulation of *il1β* and *tnf* with *A. platensis* in Nile tilapia.‏.

## Conclusion

It is evident that the growth performance, immunological responses, serum proteins, morphometry of the intestine, and antioxidant and proinflammatory cytokine gene expression of tilapia are all positively impacted by *A. platensis* and lemongrass oil alone or in combination. Moreover, the antibacterial effects of both treatments, especially against *A. hydrophila* infection. Finally, non-toxic natural remedies like *A. platensis* and lemongrass oil can be used to improve the activity’s overall sustainability and are considered eco-friendly.

## Data Availability

The datasets used and/or analyzed during the current study available from the corresponding author on reasonable request.

## Abbreviations

*A. hydrophila*: *Aeromonas hydrophila*
ALT: Alanine transaminase
ALP: Alkaline phosphatase
AST: Aspartate aminotransferase
CAT: Catalase
DNA: Deoxyribonucleic acid
FCR: Feed conversion ratio
GPx: Glutathione peroxidase
Hb: Hemoglobin
HE: Hematoxylin and eosin
IL-8: Interleukin 8
IL-1β: Interleukin 1β
LEO: Lemongrass essential oil
LG: Lemongrass
NF-κB: Nuclear factor kappa
*O. niloticus*: *Oreochromis niloticus*
PCV: Packed cell volume
PCR: Real-time polymerase chain reaction
RBCs: Red blood cells
RNA: Ribonucleic acid
AP: A. platensis
WBC: White blood cell

## Declarations

### ARRIVE guidelines

The investigation was conducted in accordance with the ARRIVE standards, as confirmed by the authors.
